# Diagnostic performance of 99mTc-HYNIC-PSMA SPECT/CT for biochemically recurrent prostate cancer after radical prostatectomy

**DOI:** 10.3389/fonc.2022.1072437

**Published:** 2022-12-07

**Authors:** Bo Li, Lili Duan, Jingqi Shi, Yunyun Han, Wei Wei, Xiaoliang Cheng, Yong Cao, Akeban Kader, Degang Ding, Xinyu Wu, Yongju Gao

**Affiliations:** ^1^ Henan Key Laboratory for Molecular Nuclear Medicine and Translational Medicine, Department of Nuclear Medicine, Henan Provincial People’s Hospital & Zhengzhou University People’s Hospital, Zhengzhou, China; ^2^ Department of Medical Imaging, Xinjiang Production and Construction Corps 13 division Red Star Hospital, Hami, China; ^3^ Department of Urology Surgery, Henan Provincial People’s Hospital & Zhengzhou University People’s Hospital, Zhengzhou, China; ^4^ Department of Pharmacy, The First Affiliated Hospital of Xi'an Jiaotong Universityl, Xi’an, China

**Keywords:** 99mTc-HYNIC-PSMA, prostate cancer, biochemically recurrent, diagnostic performance, SPECT/CT

## Abstract

**Objectives:**

99mTc-HYNIC-PSMA is a novel technetium-99m-labeled small-molecule inhibitor of prostate-specific membrane antigen (PSMA) for detection of prostate cancer. The present study investigated the diagnostic yield of 99mTc-HYNIC-PSMA Single photon emission computed tomography (SPECT)/CT in 147 patients with biochemically recurrent prostate cancer after radical prostatectomy.

**Methods:**

147 patients with biochemical relapse after radical prostatectomy were finally eligible for this retrospective analysis. The median prostate-specific antigen (PSA) level was 8.26 ng/mL (range, 0.22-187.40 ng/mL). Of the 147 patients, 72 patients received androgen deprivation therapy (ADT) at least 6 months before the 99mTc-HYNIC-PSMA SPECT/CT. All patients underwent planar whole-body scans and subsequent SPECT/CT of the thoracic and abdominal regions after intravenous injection of 705 ± 70 MBq of 99mTc-HYNIC-PSMA. Images were evaluated for the presence and location of PSMA-positive lesions, in which SUVmax were also measured. Detection rates were stratified according to PSA levels, ADT and Gleason scores. The relationships between SUVmax and clinical characteristics were analyzed using univariate and multivariable linear regression models for patients with positive findings.

**Results:**

Of the 147 patients, 99mTc-HYNIC-PSMA SPECT/CT revealed at least one positive lesion in 118 patients with a high detection rate (80.3%). The detection rates were 48.6% (17/35), 85.1% (40/47), 92.1% (35/38), and 96.3% (26/27) at PSA levels of greater than 0.2 to 2, greater than 2 to 5, greater than 5 to 10, and greater than 10 ng/mL, respectively. PSMA SPECT/CT indicated local recurrence, lymph node metastases, bone metastases, and visceral metastases in 14 (9.5%), 73 (49.7%), 48 (32.7%) and 3 (2.0%) patients. The detection rates of local recurrence and metastasis increased with increasing PSA levels. The detection rate was higher in patients treated with ADT than those without (90.3% vs. 70.7%; P =0.0029). In patients with Gleason scores ≥8, detection rate was slightly higher than those with ≤7 (81.7% vs. 78.5%), but not statistically significant (P = 0.6265). Multivariable linear regression analysis showed a significant correlation of PSA levels and ADT with SUVmax (P=0.0005 and P=0.0397).

**Conclusions:**

99mTc-HYNIC-PSMA SPECT/CT offers high detection rates for biochemically recurrent prostate cancer after radical prostatectomy. The detection rate and SUVmax were positively correlated with PSA levels and ADT.

## Introduction

Prostate cancer (PCa) is the second most common malignant tumor and the third leading cause of cancer-related death in men worldwide. Although a substantial portion of PCa can be cured by surgery or radiotherapy if detected early, recurrence or metastatic PCa remains a therapeutic challenge. Biochemical recurrence (BCR) occurs in up to 40% of PCa patients after primary treatment (1). Timely detection of disease recurrence is important for curatively intended treatment. Despite recent advances in conventional imaging, such as contrast-enhanced CT or MRI imaging, these imaging modalities exhibit limited sensitivity for recurrence assessment, especially in cases with low serum prostate-specific antigen (PSA) levels (1, 2).

In recent years, prostate-specific membrane antigen (PSMA) has received significant interest as a target for prostate cancer imaging. PSMA is a well-characterized membrane protein expressed 100- to 1000-fold higher on the surface of PCa cells than on benign prostate cells or normal tissue. Several PET agents, such as 68Ga-PSMA-11 and 18F-PSMA-1007, have been increasingly used to diagnose and guide salvage radiation therapy for biochemically recurrent PCa (3–5). A growing body of evidence suggests that PSMA PET has very high sensitivity and specificity for tumor sites in patients with BCR, even at PSA values less than 1 ng/mL (4, 6, 7). Based on its excellent diagnostic performance, several international PCa guidelines, including the European Association of Urology guideline, recommend PSMA PET/CT for patients with recurrent PSA after primary therapy (2, 8, 9).

In addition to small molecule inhibitors suitable for PET imaging, 99mTc-labeled small molecule inhibitor of PSMA has gained increasing interest recently. Given the broad availability of SPECT/CT devices, lower instrumentation and radionuclide costs, SPECT/CT systems have huge prospects for application to improve PSMA imaging capacity as more patients with prostate cancer undergo PSMA imaging. Several 99mTc-labeled PSMA inhibitors have hitherto been developed to detect PCa, including 99mTc-MIP-1404, 99mTc-MIP-1405, 99mTc-PSMA-I&S, and 99mTc-EDDA/HYNIC-iPSMA. Schmidkonz et al. (10–12) and García-Pérez et al. (13) have demonstrated the diagnostic potential of 99mTc-MIP-1404 and 99m Tc-EDDA/HYNIC-iPSMA in detecting BCR of PCa, even at low and very low serum PSA levels.

In recent years, a novel 99mTc-labeled PSMA inhibitor (HYNIC-Glu-Urea-A, 99mTc-HYNIC-PSMA) with specific accumulation in PSMA-positive tumors and low dosimetry has been synthesized (14, 15). Additionally, the labeling method of 99mTc-HYNIC-PSMA is simpler, quicker, and don’t require further purification, thus making it suitable for routine clinical applications. Nonetheless, little evidence is available on its clinical value in PCa patients with BCR. To bridge this knowledge gap, we sought to report our experience on the use of 99mTc-HYNIC-PSMA in 147 PCa patients with BCR.

## Patients and methods

### Patients

From October 2017 to March 2022, 751 patients underwent 99mTc-HYNIC-PSMA SPECT/CT at the Department of Nuclear Medicine, Henan Provincial People’s Hospital. We selected patients that satisfied the following inclusion criteria: (1) Histopathological diagnosis of PCa; (2) Completed primary treatment: radical prostatectomy with or without pelvic lymph node dissection; (3) BCR in PCa: BCR was defined as two consecutive PSA >0.2 ng/mL after radical prostatectomy (16, 17); (4) Available 99mTc-HYNIC-PSMA SPECT/CT data at the time of BCR.

Based on these criteria, 147 eligible patients were included in our study. A complete medical history and demographic data were obtained from each patient, including histologic confirmation of PCa either by needle biopsy or prostatectomy, Gleason scores, initial clinical stage, NCCN risk classification, PSA values, and prior and current PCa therapies. The clinical characteristics of enrolled patients are depicted in **Table 1**.

**Table 1:** Clinical characteristics of 147 patients.

Characteristic		Value
Age at SPECT/CT (years)		70 ± 8 (range: 49–87)
Injected dosage (MBq)		705 ± 70 (range: 495–855)
Initial therapy	RP only	128 (87.1%)
	RP and RT	19 (12.9%)
Gleason score	≤7	65 (44.2%)
	≥8	82 (55.8%)
Initial clinical stage	T1	15 (10.2%)
	T2	75 (51.0%)
	T3	38 (25.9%)
	T4	19 (12.9%)
	N0	110 (74.8%)
	N1	18 (12.3%)
	Nx	19 (12.9%)
NCCN risk group	Low	15 (10.2%)
	Intermediate	45 (30.6%)
	High	87 (59.2%)
PSA level before SPECT/CT (ng/ml)	>0.2-2	35 (23.8%)
	>2-5	47 (32.0%)
	>5-10	38 (25.8%)
	>10	27 (18.4%)
	mean ± SD	8.26 ± 17.67 (range: 0.22–187.40)
ADT	Present	72 (49.0%)
	Absent	75 (51.0%)
Localization of PSMA positive lesions*	prostate region	14 (9.5%)
	lymph node	73 (49.7%)
	bone	48 (32.7%)
	viscera	3 (2.0%)

RP, radical prostatectomy; RT, radiation therapy; ADT, androgen deprivation therapy.

*More than 1 region could be involved per patient.

This study was approved by the Ethics Committee of Henan Provincial People’s Hospital & Zhengzhou University People’s Hospital according to the principles of the Declaration of Helsinki. All patients gave written informed consent for the use of their clinical data.

### Radiosynthesis of 99mTc-HYNIC-PSMA

99mTc-HYNIC-PSMA was synthesized as previously described (14, 15). Briefly, 10 μg of HYNIC-Glu-Urea-A, 0.5 mL of EDDA (20 mg/ml in 0.1 M NaOH), 0.5 ml Tricine solution (40 mg/ml in 0.2 M PBS, pH = 6.0), 25 μl of SnCl_2_ solution (1 mg/ml in 0.1 M HCl) and 1110–2220 MBq of Na^99m^TcO_4_ were heated for 15 min at 100°C. The radiochemical purity was not less than 95%, as determined by radio-TLC and high-performance liquid chromatography (HPLC).

### Image acquisition and reconstruction

All patients underwent 99mTc-HYNIC-PSMA SPECT/CT 3-4 h after intravenous injection of 10 MBq/kg of 99mTc-HYNIC-PSMA. Planar whole-body (WB) images and SPECT/CT data acquisition were performed using a Symbia T16 SPECT/CT scanner (Siemens Symbia Intevo, Erlangen, Germany) that combines a dual-headed gamma camera with a 16-slice CT system. WB planar scintigraphy (anterior and posterior) was acquired using a low-energy high-resolution (LEHR) collimator with a matrix of 256 x 1024 and a scan speed of 12 cm/min. Following planar images acquisition, SPECT/CT of the thoracic and abdominal regions was performed for each patient. SPECT/CT was performed to maximize sensitivity, given that it is more sensitive for detecting subsequent lesions than planar imaging. Imaging parameters for SPECT were: 6 degrees angular resolution and 30s per step with a 256 × 256 matrix. The low-dose CT scan parameters were: 130 kV and 25 reference mAs modulation. CT data were reconstructed at a 5-mm slice thickness using B31s medium smooth kernels (Siemens Healthineers). SPECT components were calibrated monthly using a ^57^Co source (Calibrated Sensitivity Source) for quantitative imaging.

SPECT data were reconstructed using Flash3D with scatter and attenuation corrections. The quantitative uptake maps were reconstructed using xSPECT Quant (Siemens, Germany), which corrected attenuation and scatter and provided standardized calibrations for absolute quantification.

### Image analysis

SPECT, CT, and fused imaging of 99mTc-HYNIC-PSMA scan were analyzed with dedicated software (Syngo, Siemens Medical Solutions USA, Inc. and Toshiba Corp.) SPECT/CT images were reviewed by two experienced nuclear medicine physicians, and a final diagnosis was achieved by consensus. Both nuclear medicine physicians were blinded to the clinical information. Positive lesions were identified if the 99mTc-HYNIC-PSMA uptake in the lesion was higher than the surrounding normal tissues and not associated with physiological uptake. In this study, all lesions suggesting recurrence of PCa were categorized into local recurrence, lymph node metastases, bone metastases, and visceral metastases (e.g., lung, liver).

For quantitative analysis of positive lesions, volume-of-interest (VOI) was delineated around each target lesion on the fused SPECT/CT images using syngo, setting isocontours at 40% of maximum uptake. Uptake intensity was expressed as the maximum standardized uptake value (SUVmax) based on the measured activity concentrations of tissues normalized by patient weight and injected activity. The highest SUVmax was selected for quantitative assessment for patients with multiple positive lesions. In addition, the maximum diameter in the transaxial plane of 99mTc-HYNIC-PSMA positive lymph nodes was determined based on the CT images from the hybrid SPECT/CT.

### Statistical analysis

The detection rates were plotted against the absolute PSA levels. Mann–Whitney U tests were used to evaluate differences between single groups and to evaluate differences in PSA levels. The relationships between SUVmax and PSA, ADT, GS, age and injected dosage were analyzed using univariate and multivariable linear regression models. A P-value<0.05 was statistically significant. Statistical analyses were performed using R software (version 3.5.3).

## Results

### Radiosynthesis and quality control of 99mTc-HYNIC-PSMA

The radiopharmaceutical ^99m^Tc-HYNIC-PSMA was obtained at a radioactivity concentration of 341 ± 63 MBq/mL (9.2 ± 1.7 mCi/mL; referred to the time of expiry), with a high radiochemical purity of 97.5 ± 1.2%. In our study cohort, the mean injected activity of 99mTc-HYNIC-PSMA was 705 ± 70 MBq (19.1 ± 1.9 mCi; range 495–855 MBq). No adverse events or clinically detectable pharmacologic effects were observed in enrolled patients after intravenous injection of 99mTc-HYNIC-PSMA.

### Detection efficacy

**Figure 1:**
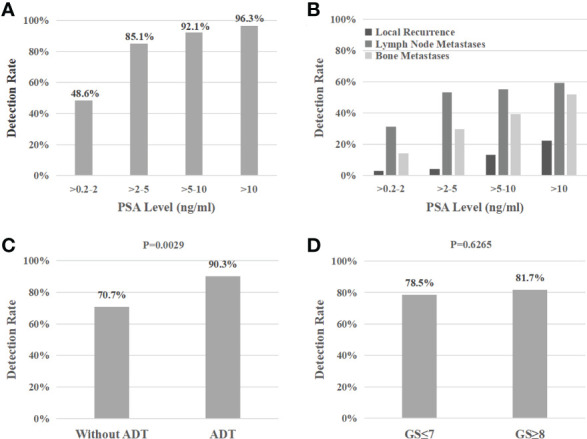
Detection rate of 99mTc-HYNIC-PSMA SPECT/CT based on PSA levels **(A)**, different recurrence regions **(B)**, androgen deprivation therapy **(C)**, and Gleason scores **(D)**.

During the study, at least one PSMA-positive lesion was detected in 80.3% (118/147) of patients. The detection rate of 99mTc-HYNIC-PSMA SPECT/CT was 48.6% (17/35), 85.1% (40/47), 92.1% (35/38), and 96.3% (26/27) at PSA levels of >0.2-2 ng/ml, >2-5 ng/ml, >5-10 ng/ml, and >10 ng/ml, respectively. As shown in **Figure 1A**, the detection rate increased with higher serum PSA levels. In addition, patients with negative 99mTc-HYNIC-PSMA SPECT/CT findings had significantly lower PSA levels than those with positive findings (2.15 ± 2.80 vs. 9.76 ± 19.39ng/mL; P < 0.001) (**Table 2**).

**Table 2:** The average PSA-values in this study population considering 99mTc-HYNIC-PSMA SPECT/CT results, androgen deprivation therapy, and Gleason scores.

Categories	PSA(ng/mL)	P value
Negative vs. Positive SPECT/CT findings	2.15 ± 2.80/0.22-12.89 (n=29) vs. 9.76 ± 19.39/0.23-187.40 (n=118)	P<0.0001
Without ADT vs. ADT	6.16 ± 10.83/0.22-87.36 (n=75) vs. 10.44 ± 22.59/0.23-187.40 (n=72)	P=0.0423
GS ≤ 7 vs. GS≥8	7.85 ± 12.16/0.22-87.36 (n=65) vs. 8.59 ± 21.11/0.23-187.40 (n=82)	P=0.9907

Date are median ± SD/range.

Furthermore, the detection rate of patients treated with androgen deprivation therapy (ADT) was significantly higher than those without (90.3% vs. 70.7%; P=0.0029) (**Figure 1C**). However, the PSA values were significantly different between the two groups (10.44 ± 22.59 vs. 6.16 ± 10.83 ng/mL; P=0.0423) (**Table 2**), which exerted a confounding effect on the assessment of detection rates.

99mTc-HYNIC-PSMA SPECT/CT was positive in 78.5% (51/65) of patients with Gleason scores ≤7 and 81.7% (67/82) of patients with Gleason scores ≥8 (P = 0.6265) (**Figure 1D**). Similarly, the mean PSA levels were comparable between both groups (7.85 ± 12.16 vs. 8.59 ± 21.11 ng/mL; P=0.9907) (**Table 2**).

### PSMA-positive lesions location

**Figure 2:**
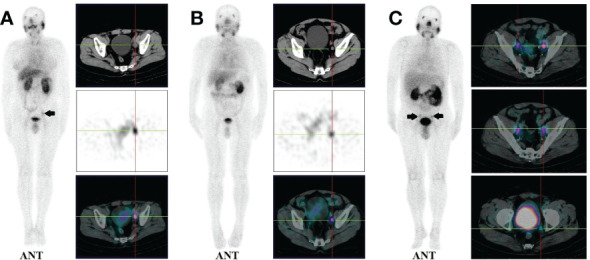
**(A)**: Images of biochemically recurrent prostate cancer in a 72-year-old male with rising PSA levels after radical prostatectomy (PSA, 3.43 ng/ml; Gleason score, 4 + 4). Whole-body planar scintigraphy (pointed by black arrow) and transaxial SPECT/CT fusion images indicated a diameter of 13 mm lymph node metastases near the left iliac blood vessels, which shows pathological tracer uptake (SUVmax, 3.67). Final pathology confirmed prostate cancer metastasis. **(B)**: Images of biochemically recurrent prostate cancer in a 61-year-old male with rising PSA levels after radical prostatectomy (PSA, 0.73 ng/ml; Gleason score, 4 + 5). Transaxial SPECT and fused SPECT/CT images show high 99mTc-HYNIC-PSMA uptake (SUVmax, 2.74) in subcentimeter (8 mm) lesions, as determined by corresponding CT. The patient underwent radiation therapy, and his PSA level decreased to 0.11 ng/ml. **(C)**: Images of biochemically recurrent prostate cancer in a 70-year-old male with rising PSA levels after radical prostatectomy (PSA, 9.26 ng/ml; Gleason score, 4 + 4). Whole-body planar scintigraphy (pointed by black arrows) and transaxial SPECT/CT fusion images show multiple lymph node metastases (diameter between 7 and 12 mm) near the iliac blood vessels, which shows pathological tracer uptake (the highest SUVmax, 3.42). The patient received ADT, and his PSA level decreased to 0.53 ng/ml.

**Figure 3:**
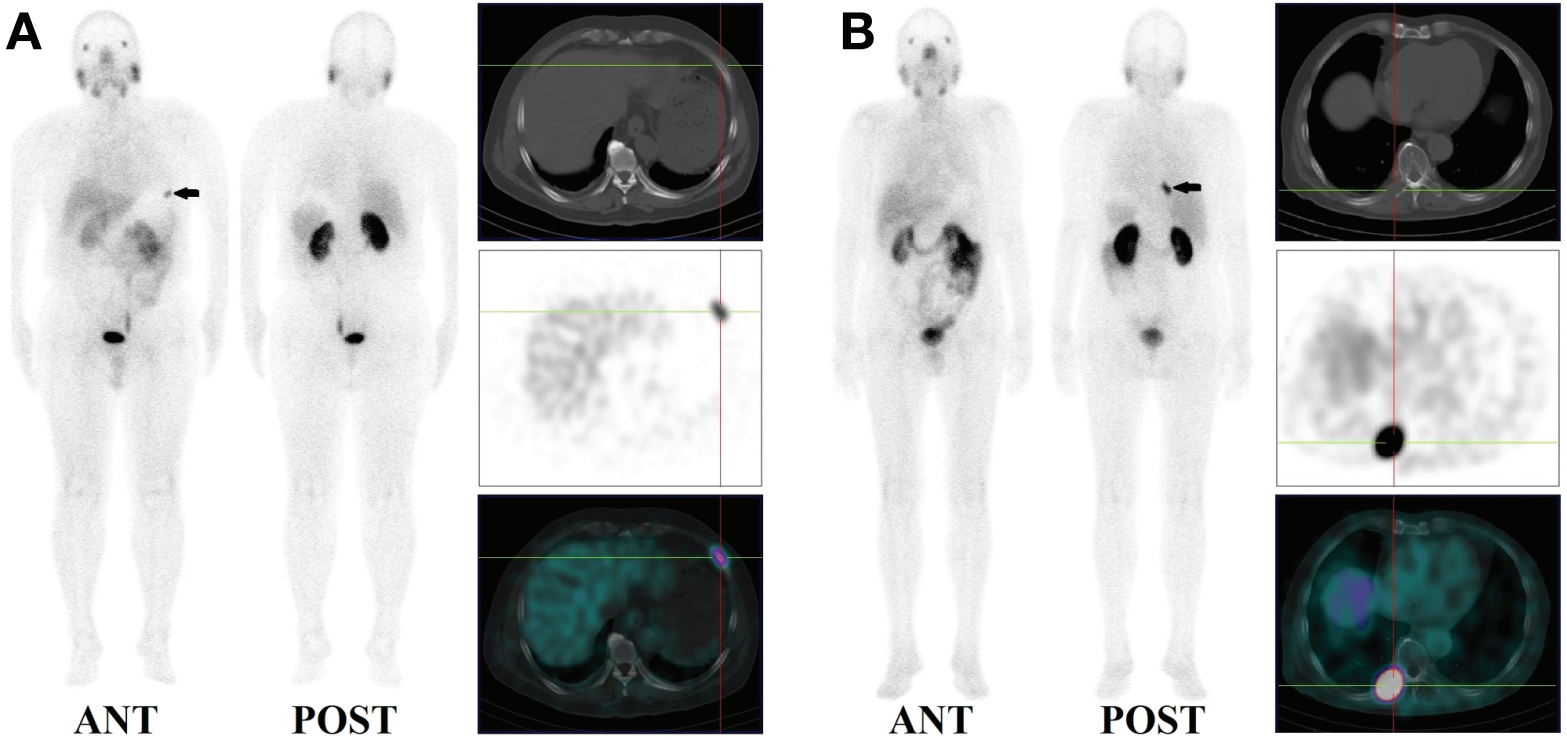
**(A)**: Images of biochemically recurrent prostate cancer in a 71-year-old male with increasing PSA levels after radical prostatectomy (PSA, 0.41 ng/ml; Gleason score,3+4). Whole-body planar scintigraphy (pointed by black arrow) and fused SPECT/CT images show high 99mTC-HYNIC-PSMA uptake (SUVmax, 2.97) in the left 6th rib. However, the corresponding CT showed no obvious bone destruction. The patient received ADT, and his PSA levels decreased to 0.10 ng/ml. **(B)**: Images of biochemically recurrent prostate cancer in a 59-year-old male with increasing PSA levels after radical prostatectomy (PSA, 6.30 ng/ml; Gleason score, 5 + 5). Whole-body planar scintigraphy (pointed by black arrow) and fused SPECT/CT images show high 99mTC-HYNIC-PSMA uptake (SUVmax, 8.63) in the right 8th rib, the corresponding CT showed obvious bone destruction. The patient received radiation therapy and his PSA level decreased to 0.31 ng/ml.

**Figure 4:**
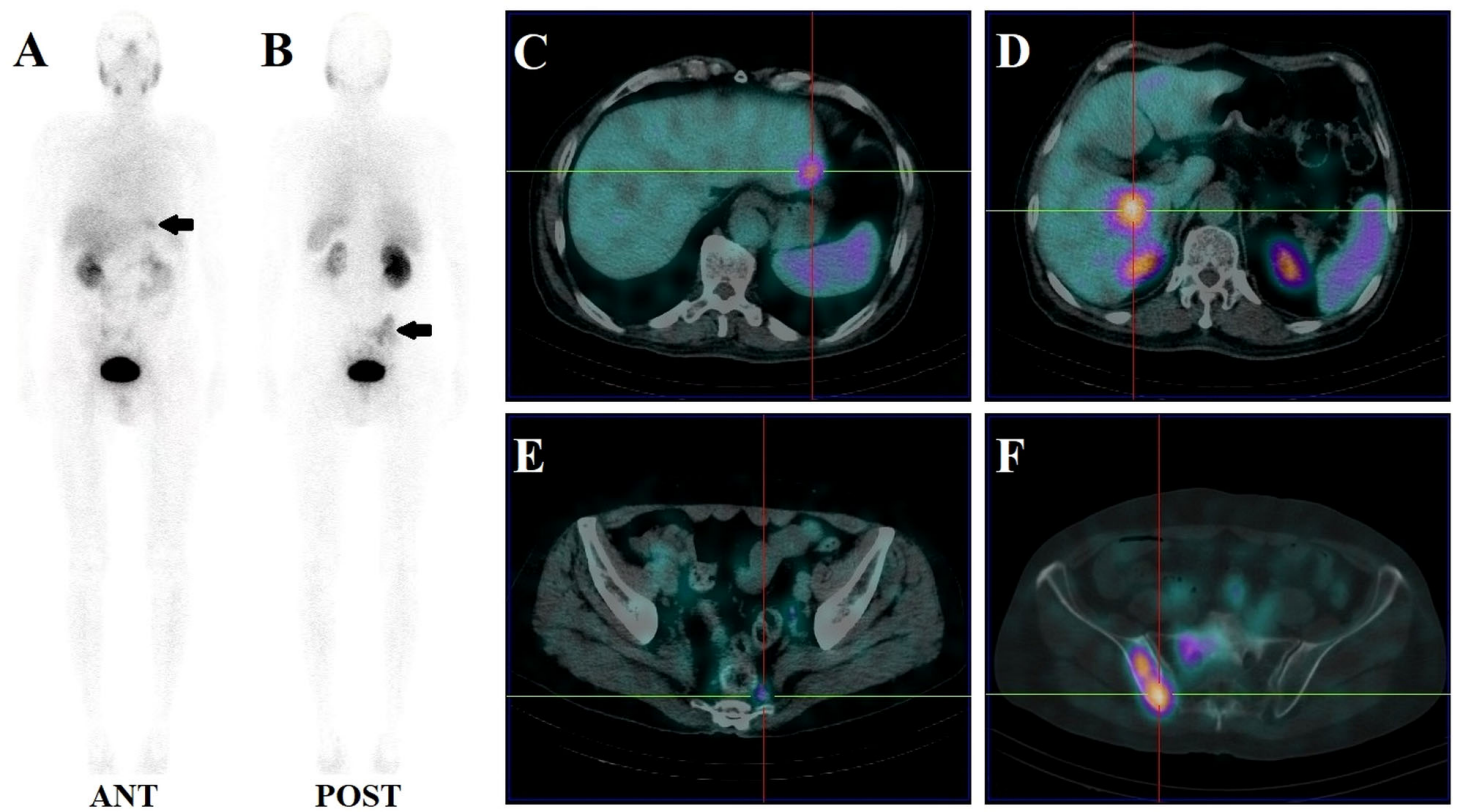
Images of biochemically recurrent prostate cancer in a 70-year-old male with increasing PSA levels after radical prostatectomy (PSA, 187.40 ng/ml; Gleason score, 4 + 5). Whole-body planar scintigraphy (**A, B**; pointed by black arrows) and fused SPECT/CT images show multiple metastases in the liver (**C, D**; the highest SUVmax, 4.61), pelvic lymph node (**E**; SUVmax, 1.84), and sacrum and right iliac bone (**F**; SUVmax, 6.82). The patient received chemotherapy and his PSA level decreased to 23.73 ng/ml.

14 patients (9.5%) had local recurrence in the prostate region, 73 patients (49.7%) had lymph node metastases (representative example in **Figure 2**), 48 patients (32.7%) had bone metastases (representative example in **Figure 3**), 2 patients had lung metastases, and multiple organ metastases (lymph node, liver and bone) were present in 1 patient (representative example in **Figure 4**). **Table 1** lists the different regions where disease recurrence occurred.

Local recurrence were observed in 2.9% (1/35), 4.3% (2/47), 13.2% (5/38), and 22.2% (6/27) cases with PSA levels of >0.2-2, >2-5, >5-10, and >10 ng/mL, respectively, suggesting elevated local recurrence rates at higher PSA levels.

As illustrated in **Figure 1B**, lymph node metastasis was detected in 31.4% (11/35), 53.2% (25/47), 55.3% (21/38), and 59.3% (16/27) of patients with PSA levels of 0.2-2, 2-5, 5-10, and >10 ng/mL, respectively. Pelvic lymph node metastases were present in 17.4% (4/23) of patients with early BCR (PSA levels of less than 1 ng/mL) (representative example in **Figure 2B**). Additionally, CT images obtained during SPECT/CT examination were used to measure the diameter of PSMA-positive lymph nodes. 99mTc-HYNIC-PSMA detected 35.6% (26/73) of PSMA-positive lymph nodes with a diameter less than 10 mm (representative examples in **Figures 2B, 2C**).

Moreover, 13.0% (3/23) of bone metastases were found in patients with PSA levels below 1ng/ml (**Figure 3A**). 14.3% (5/35), 29.8% (14/47), 39.5% (15/38), and 51.9% (14/27) of patients with PSA levels of 0.2-2, 2-5, 5-10, and >10 ng/mL, respectively, presented with bone metastases (**Figure 1B**). Besides, 165 positive bone lesions were detected (4 patients with diffuse bone metastases were excluded), of which 57 (34.5%) were visible on 99mTc-HYNIC-PSMA SPECT but not on CT (see **Figure 3A** for a representative example).

Lung metastases were observed in 2 patients with PSA levels of 7.30 and 18.27 ng/ml, respectively. As shown in **Figure 4**, only one patient with a PSA level of 187.40 ng/ml had liver metastases.

### Influence of clinical characteristics on SUVmax

The relationships between SUVmax and clinical characteristics (PSA, ADT, GS, Age and Injected dosage) were analyzed using univariate and multivariable linear regression models for patients with 99mTc-HYNIC-PSMA positive findings. The univariate linear regression analysis showed that SUVmax had a significant correlation with PSA, ADT, and GS (P=0.0002, P=0.0456 and P=0.0444, respectively) but no significant correlation with age and injected dosage (P=0.3199 and P=0.1219, respectively) (**Table 3**). In addition, multivariable linear regression analysis showed a significant correlation of PSA and ADT with SUVmax (P=0.0005 and P=0.0397, respectively) (**Table 3**).

**Table 3:** Univariate and multivariable linear regression analyses: Influence of Clinical characteristics on SUVmax.

IV	RC	95%CI		P value	RC	95%CI		P value
		Lower	Upper			Lower	Upper	
PSA	2.162	1.049	3.274	0.0002	0.048	0.022	0.074	0.0005
ADT	0.031	0.001	0.060	0.0456	1.097	0.053	2.141	0.0397
GS	0.066	0.002	0.130	0.0444	0.364	-0.122	0.849	0.1405
Age	-0.242	-0.721	0.238	0.3199	-0.058	-0.123	0.007	0.0808
Injected dosage	3.404	-0.922	7.730	0.1219	0.006	-0.001	0.013	0.1094

IV, independent variables; RC, regression coefficient; CI, confidence interval.

## Discussion

An increasing body of evidence suggests that progression to metastasis or recurrence of PCa remains an important cause of death (1, 18). The early detection of prostate cancer recurrence following primary therapy is crucial to improve patient outcomes. It has been established that conventional imaging modalities (bone scan, CT, and MRI) exhibit limited ability to detect recurrence accurately, especially at low PSA levels (2). Therefore, an imaging technique that can detect recurrences effectively is urgently needed. Given that PSMA is overexpressed predominantly in 90-100% of PCa lesions, it has gained increasing attention as an attractive target for diagnosis. There is a rich literature available substantiating that 68Ga and 18F labeled PSMA PET-CT imaging is a promising modality for detecting PCa recurrence (3, 4, 6, 7, 19). To evaluate the diagnostic potential of 99mTc-HYNIC-PSMA in PCa recurrence, we retrospectively analyzed the clinical and imaging data from 147 patients with biochemical recurrence of PCa. We provided compelling evidence that 99mTc-HYNIC-PSMA SPECT/CT could effectively detect and localize PSMA-positive lesions with an overall detection rate of 80.3% (118/147). Detection rates were significantly increased at PSA levels above 2 ng/mL (90.2% vs. 48.6%; P < 0.0001). In addition, univariate and multivariable linear regression analysis showed that SUVmax positively correlated with PSA levels, which could potentially reflect disease activity.

Over the years, 99mTc-labeled PSMA inhibitors have been reported to detect BCR (See the summary of studies in **Table 4**), although limited studies have been conducted. Two studies by Schmidkonz and colleagues reported that the overall detection rates of 99mTc-MIP-1404 for BCR of PCa were 77% (174/225) (10) and 70% (42/60) (12), respectively. Similarly, Su et al. (20) and Liu et al. (21) reported detection rates of 78% (39/50) and 73% (151/208) for 99mTc-HYNIC-PSMA in the localization of BCR. A detection rate of 57% (87/152) was reported for 99mTc-PSMA-I&S, which is lower than our finding (22). A prospective study reported a lower detection rate (58%, 21/36) using the new radiopharmaceutical 99mTc-PSMA-T4 (23).

**Table 4:** Summary detection rate of series reporting 99mTc-labled PSMA for biochemical recurrence of PCa.

Imaging modality	Study Design	DR	PSA stratified DR (ng/ml)		Reference
99mTc-PSMA-T4	Prospective	21/36 (58%)	NR		23
99mTc-PSMA-I&S	Retrospective	87/152 (57%)	≤1 >1-4 >4-10 >10	8/41 (20%) 32/58 (55%) 29/35 (83%) 18/18 (100%)	22
99mTc-MIP-1404	Retrospective	25/50 (50%)	>0.2-0.5 >0.5-1	11/25 (44%) 14/25 (56%)	11
	Retrospective	174/225 (77%)	≤1 >1-3 >3-5 >5-10 >10-20 >20	25/43 (58%) 38/61 (62%) 28/33 (85%) 33/37 (89%) 29/29 (100%) 21/22 (96%)	10
	Retrospective	42/60 (70%)	≤1 >1-2 >2-5 >5-10 >10-20 >20	4/11 (36%) 6/14 (43%) 13/15 (87%) 6/6 (100%) 5/5 (100%) 8/9 (89%)	12
99mTc-HYNIC-PSMA	Retrospective	39/50 (78%)	≤1 >1-4 >4-10 >10	3/10 (30%) 8/10 (80%) 5/5 (100%) 23/23 (100%)	20
	Retrospective	151/208 (73%)	>0.2-1 >1-2 >2-5 >5-10 >10	28/55 (51%) 14/23 (61%) 44/53 (83%) 39/45 (87%) 26/32 (81%)	21
	Retrospective	118/147 (80%)	>0.2-2 >2-5 >5-10 >10	17/35 (49%) 40/47 (85%) 35/38 (92%) 26/27 (96%)	*Present study*

DR, detection rate; NR, not reported.

Interestingly, Schmidkonz et al. and Reinfelder et al. observed 54% and 40% detection rates for PSA levels less than 2 ng/mL (10, 12). A similar detection rate of 54% was reported by Liu et al. (21) by analysis of 78 patients with PSA levels less than 2 ng/mL, slightly higher than our findings. Moreover, we found that the detection rate was 40.0% (4/10) and 30.8% (4/13) at PSA levels of 0.5-1 ng/ml and 0.2-0.5 ng/ml. Schmidkonz et al. reported a favorable detection rate of 56% and 44% at low (0.5-1 ng/ml) and very low (0.2-0.5 ng/ml) PSA levels, respectively (11). Nevertheless, 2 studies reported less promising detection rates at PSA levels less than 1 ng/ml (30% and 20% with 99mTc-HYNIC-PSMA and 99mTc-PSMA-I&S, respectively) (20, 22).

An increasing body of evidence suggests that 68Ga-PSMA ligand, a dedicated PET agent, can significantly increase the detection rate of recurrent PCa (24–29). The overall detection rate ranged from 47% to 97%, and at PSA levels above 2 ng/ml, a detection rate ranging from 75% to 97% was observed, while a detection rate of 80% was found in our study. For PSA levels less than 2 ng/mL, a 61% to 69% detection rate was reported, 15% higher than reported in the present study. Furthermore, several studies reported promising detection rates at low PSA levels, ranging from 58% to 75% at 0.5-1 ng/ml and 38% to 62% at <0.5 ng/ml (27, 29–32), which are higher than observed in our study. However, Meredith et al. (33) reported a significantly lower detection rate in patients with low PSA levels. The detection rate was 25% at PSA levels 0.5-1 ng/mL and 18% at PSA levels less than 0.5 ng/mL. The primary advantage of 68Ga-PSMA PET is its relatively high detection rate at low serum PSA levels. It is widely acknowledged that compared with SPECT, PET has the advantages of higher sensitivity and higher spatial resolution when evaluating small lesions associated with low PSA levels. A direct comparison involving the same patient population between 68Ga-PSMA and 99mTc-PSMA was reported by Fallahi et al. (34). They found that lesion size strongly influenced SPECT detection of lesions.

Herein, we showed that the detection rate and SUVmax from patients treated with ADT were significantly high. Consistent with our findings, Einspieler et al. (35) and Afshar-Oromieh et al. (27, 30) demonstrated a strong positive correlation between ADT and 68Ga-PSMA Ligand PET/CT positivity. However, Eiber et al. (29) found no significant difference in detection rates regarding ADT. Indeed, ADT is highly controversial in regards to PSMA ligand uptake. Short-term ADT is known to increase PSMA expression in PCa cells, thereby increasing PSMA ligand uptake (36). In contrast, it has been reported that PSMA uptake decreases with long-term ADT, decreasing the sensitivity of PSMA PET for detecting PCa lesions (37, 38). Furthermore, long-term ADT also reduces tumor cell numbers. Thus, the higher detection rate in patients who received at least 6 months of ADT may be attributed, to a certain extent, to have more advanced-stage disease. The present study found that patients with ADT had a higher percent of advanced disease than those without ADT (81.9% vs. 68.0%), but the difference was not statistically significant (p=0.0526). Accordingly, PSA levels were higher in patients with ADT than in those without such therapy.

The present study found no significant difference between the detection rates at different Gleason scores, consistent with the literature (30, 39). In addition, a significant positive correlation between Gleason scores and SUVmax was observed during univariate linear regression analysis but not during multivariate regression analysis. Schmidkonz et al. found a significant correlation between the PSMA PET positivity and the Gleason score, which was attributed to PSMA upregulation at higher Gleason scores (10, 40).

99mTc-HYNIC-PSMA can detect recurrent lesions in different locations, such as local recurrence, lymph nodes, bone metastases, and visceral metastases. Some patients presented multiple lesions at different sites. The detection rates of 9.5%, 49.7%, 32.7% and 2.0% in the prostate, lymph nodes, bone and visceral regions, respectively. The lymph nodes are one of the most common sites of PCa recurrence after primary therapy, especially in the pelvic region. Pelvic lymph node metastases were detected in 17.4% of patients with early BCR (PSA levels of less than 1 ng/mL). Notably, 99mTc-HYNIC-PSMA detected 35.6% PSMA positive lymph nodes less than 10 mm in diameter, highlighting good detection yield for small lymph node metastases, suggesting that SPECT can be used to diagnose lymph node metastases although it has an inferior resolution than PET.

In addition, 165 PSMA-positive bone lesions could be detected on 99mTc-HYNIC-PSMA SPECT. Of these, 34.5% of PSMA-positive bone lesions were recognized only by SPECT but not by CT. A potential explanation could be that the 99mTc-HYNIC-PSMA SPECT detects metabolic changes in bone tissue during the development of bone metastases at a very early stage, whereas a CT scan shows no obvious soft tissue or bone destruction (41). Notably, early detection of lymph node and bone metastases is beneficial to PCa patients since early treatment of BCR results in better outcomes (42).

This retrospective analysis has certain limitations. The pathological confirmation of positive lesions was available in only 19 patients. Accordingly, it is highly conceivable that false-positive lesions were detected. Nevertheless, the PSA levels decreased in 68 patients with positive imaging results who were referred for follow-up treatment, indicating the PSMA-positive lesions were most likely true positives. Sergieva et al. (23) demonstrated a sensitivity of 84.37% (27/32), a specificity of 84.37% (27/32) and an accuracy of 86.11% (31/36) for detecting BCR using 99mTc-PSMA-T4 in 36 PCa patients. Therefore, it is necessary to perform further analyses to evaluate the sensitivity and specificity of 99mTc-HYNIC-PSMA imaging in detecting BCR. In addition, the present study was retrospective, limiting our ability to obtain more comprehensive data regarding patient treatment and follow-up. Accordingly, prospective studies are warranted to validate our findings.

## Conclusion

The high prevalence of prostate cancer emphasizes the need to develop a novel, cost-effective, and easily synthesized 99mTc-labelled PSMA ligand to leverage SPECT’s broad availability. 99mTc-HYNIC-PSMA may fill this clinical need, yielding a high detection rate for 147 PCa patients with biochemical relapse. Further analysis indicated that the detection rate and SUVmax were positively associated with increasing PSA levels and ADT.

## Data availability statement

The original contributions presented in the study are included in the article/supplementary material. Further inquiries can be directed to the corresponding authors.

## Ethics statement

The studies involving human participants were reviewed and approved by the ethics committee of Henan Provincial People’s Hospital & Zhengzhou University People’s Hospital according to the principles of the Declaration of Helsinki. All patients gave written permission for the use of their data and informed consent for the anonymous publication. The patients/participants provided their written informed consent to participate in this study.

## Author contributions

YG, XW, DD and BL designed the retrospective study. BL, XW, LD, JS, WW and YG collected and analyzed the data. YH, XC, AK and YC analyzed the data. BL, YG and XW drafted the manuscript. YG and BL can authenticate all raw data. All authors contributed to the article and approved the submitted version.
